# A Critical Review of the Prospect of Integrating Artificial Intelligence in Infectious Disease Diagnosis and Prognosis

**DOI:** 10.1155/ipid/6816002

**Published:** 2025-03-06

**Authors:** Shuaibu Abdullahi Hudu, Ahmed Subeh Alshrari, Esra'a Jebreel Ibrahim Abu-Shoura, Amira Osman, Abdulgafar Olayiwola Jimoh

**Affiliations:** ^1^Department of Basic and Clinical Medical Sciences, Faculty of Dentistry, Zarqa University, Zarqa 13110, Jordan; ^2^Department of Medical Laboratory Technology, Faculty of Applied Medical Science, Northern Border University, Arar 91431, Saudi Arabia; ^3^Department of Histology and Cell Biology, Faculty of Medicine, Kafrelsheikh University, Kafr El Sheikh, Egypt; ^4^Department of Pharmacology and Therapeutics, Faculty of Basic Clinical Sciences, College of Health Sciences, Usmanu Danfodiyo University, Sokoto 840232, Sokoto State, Nigeria

**Keywords:** artificial intelligence, diagnosis, infectious diseases, machine learning, prognosis

## Abstract

This paper explores the transformative potential of integrating artificial intelligence (AI) in the diagnosis and prognosis of infectious diseases. By analyzing diverse datasets, including clinical symptoms, laboratory results, and imaging data, AI algorithms can significantly enhance early detection and personalized treatment strategies. This paper reviews how AI-driven models improve diagnostic accuracy, predict patient outcomes, and contribute to effective disease management. It also addresses the challenges and ethical considerations associated with AI, including data privacy, algorithmic bias, and equitable access to healthcare. Highlighting case studies and recent advancements, the paper underscores AI's role in revolutionizing infectious disease management and its implications for future healthcare delivery.

## 1. Introduction

Artificial intelligence (AI) is a multidisciplinary field responsible for automatizing tasks requiring human intelligence, and it is changing life in different ways [1]. The basic classification is the ability of computers to carry out activities intended for humans [2]. AI has been instrumental in shaping scientific and technical disciplines, such as robotics, image recognition, natural language, and expert systems [3]. On the other hand, although AI is an excellent achievement for humans, it has a limit in the ability to understand organisms' lives [4]. The AI technologies that are increasingly important in managing infectious diseases provide innovative insights for various healthcare challenges. These technologies include machine learning (ML) algorithms that can process large volumes of data for pattern detection and epidemic forecasting while being implemented in diagnostic tools to detect diseases in their early stages with high accuracy, leading to improved patient outcomes [5]. Natural language processing (NLP) enables the extraction, integration, and summarization of knowledge from large volumes of medical literature as well as from electronic health records (EHRs) and social media to assist in tracking disease outbreaks and monitoring public health trends [6]. Computer vision imaging techniques are used in infection determination and disease monitoring; AI-powered imaging tools detect pneumonia in chest X-rays. Predictive analytics helps to predict the spread of infectious diseases by taking into consideration the climate, population density, and travel patterns, which helps in planning and implementing preventive measures [7]. Robotics and automation are utilized in various ways, such as disinfecting hospitals where infection risks are high, ensuring the hygienic supply of medications, and assisting in surgeries. These applications help reduce the transmission of infections [8].

The use of AI is expected to bring benefits to every field in science or information systems; however, it is dangerous if it is used irresponsibly when it is not backed by proper science [9]. The role of AI in medicine has also grown significantly, which involves the evolution of ML, deep learning, expert systems, and intelligent robotics [10]. These technologies have been used in intelligent screening, diagnosis, risk prediction, and attention-based treatment [11]. The novelty of AI in medicine is found in the methodological and conceptual content, focusing on AI and computer science [11, 12]. The methodological paper contains the science of solving the problem using the strategy, while the theoretical paper delves into the underlying issues and understanding within a medical or controlled testing environment [13]. AI in medicine has started shaping healthcare and medical sectors. AI is used in healthcare in preemptive, diagnostic, and treatment-based ways. Preventive methods employ predictive analytics to realize disease outbreaks and recognize high-risk populations, and health surveillance via wearables and applications monitors early indicators of disease [14]. Diagnostic methods include ML algorithms to interpret medical images and genomic information and NLP to gather information from medical records [15]. Many treatment-based approaches are being proposed, for example, robotics and automation for surgeries and hospital tasks and genomic sequencing so that we can better understand pathogens to make targeted treatments and vaccines [16]. Such AI applications facilitate prevention, assist diagnostic accuracy, and better treatment outcomes.

The rates of infectious disease reflect the ongoing struggle that human populations face against endemic diseases and point to a new realm for AI intervention in response. In recent years, the number of infectious diseases due to the emergence of multidrug-resistant (MDR) bacteria has increased [17]. In August 2023, over 770 million cases and at least 6.9 million deaths had been associated with the COVID-19 pandemic globally [18] which highlighted substantial reliance on previous modeling strategies that cannot cope at large especially in real-time surveillance as well predictive modeling. About 10.6 million people globally struggle with tuberculosis (TB), which will kill 1.6 million by that year but could be avoided using a vaccine. AI algorithms, represented as models such as BlueDot and HealthMap, have notably improved outbreak pattern detection by linking various data streams with more complex analytical methods [19–21]. By analyzing massive datasets that include EHRs, social media, and mobile-health datasets, these systems can pick up potential outbreak signs almost as quickly as any person along with risk assessment using ML and deep learning [22, 23]. AI can predict when outbreaks are likely to occur and where resources should be shipped, which is useful for malaria, a disease that caused 247 million cases and 619,000 deaths in sub-Saharan Africa last year [24]. This is evidenced by the 1.27 million lives lost globally to antimicrobial resistance (AMR) in 2019 [25], a stark illustration of why we need sustainable AI-based platforms that can detect and design antibiotics as computationally assisted agents with screen alerts for resistant strains difficult-to-catch through purely human-driven efforts until now. AI-based early detection, diagnostics, and precision treatment will be powerful enablers for healthcare transformation. They can save millions with more timely response during pandemics. Bringing efficiency in resource utilization as well as efficacy for public health response promises improved healthcare outcomes alongside making healthcare systems so much more resilient globally.

Infectious disease management relies on traditional methods to control and mitigate the spread of diseases. However, they have many shortcomings and are not truly effective for emerging or re-emerging infectious diseases. This is due to poor response times (such as manual collection and analysis of data), lack of strong predictability models that are not able to adapt fast enough in very dynamic environments with new pathogens, and limited human resources for surveillance/diagnosis or contact tracing. Second, traditional methods can be hampered by data overload and difficulty in integrating several types of information from various sources for real-time decision making. These concerns are escalated with globalization and added mobility, as the challenges of cross-border disease spread become too complex for traditional systems to deal with in conjunction toward more conforming regional responses. There are several ways AI integration addresses these challenges, including speeding up data analysis, making predictive models work better, and automating resource-intensive tasks such as integrating disparate streams of data. Considering the advances in AI, there are opportunities to navigate data, streamline resources, and improve decisions, which is particularly relevant for healthcare in low-resource environments. It analyzes vast amounts of data to uncover trends, guides decision making, and drives resource allocation. AI-fueled telemedicine reduces the pressure on rural and underserved healthcare facilities. Telemedicine powered by AI reduces physical visits [26]. Predictive models allow the anticipation of disease outbreaks and patient needs in advance allowing before the actual outbreaks of disease to proactively manage the resources [27], freeing healthcare professionals from administrative tasks to concentrate on patient care. AI enhances the distribution of medical supplies, respiratory aids, and staff resources, enabling effective use of resources. In general, AI increases efficiency, cuts costs, and enhances healthcare delivery, mainly within resource-limited environments.

This review outlines the critical role of AI in advancing the diagnosis and management of infectious diseases. Despite considerable progress in traditional diagnostic and prognostic methods, challenges such as delayed detection, limited accuracy, and slow response times persist. AI technologies offer promising solutions by analyzing vast amounts of medical data, including clinical symptoms, laboratory results, and imaging data, to enhance early detection and personalized treatment strategies. Recent advancements in AI, including ML algorithms and predictive models, have demonstrated the potential to address these limitations. This paper aims to explore the integration of AI in infectious disease management, evaluate its benefits, and address associated challenges. By examining the current landscape and outlining future directions, the introduction sets the stage for a comprehensive discussion on the transformative potential of AI in this critical area of the public.

## 2. Early Detection and Diagnosis

The treatment of infectious diseases relies heavily on early detection, which creates an opportunity for intervention to prevent complications, lower transmission rates, and improve patient outcomes by discovering infections early, and healthcare professionals can devise the right treatments and preventative controls, allowing diseases to be contained. Moreover, early detection allows for optimal resource allocation, deploying medical resources and personnel effectively. It is this proactive strategy that directs epidemic prevention and subsequent public health responses.

### 2.1. Key Applications of AI in Diagnosis

AI is revolutionizing the healthcare field by improving the diagnosis and treatment of infectious diseases. By analyzing large datasets through advanced algorithms, AI enhances accuracy, efficiency, and speed for early disease detection and timely intervention, which in turn reduces disease progression and transmission. AI tools analyze clinical data, predict disease outbreaks, and help allocate resources in the process of identifying hotspots of infections [28]. Portable diagnostic tools and AI-based analysis of medical imaging and genomic data allow for decentralized testing as well as pathogen identification. Incorporation into EHRs simplifies diagnostics, and real-time monitoring systems identify and monitor infections [29]. There is also accelerating testing for drug resistance through AI to direct effective treatment against and help combat AMR [20, 30]. Collectively, these applications enhance patient care, optimize resources, and strengthen public health efforts.

### 2.2. Role of AI in Early Detection

AI algorithms can sift through massive amounts of medical data on symptoms, epidemiological data, lab results, and imaging scans to recognize patterns emanating from infectious diseases. Early discovery helps end potential reservoirs and slow down or stop the transmission of pathogens to other people and helps rescue those already sick. The use of AI through automatic scanning of open-source data to identify danger signals at the outset of outbreaks is critical for improving early detection and diagnosis [31]. AI-driven component-based methodology has long been a mainstay of diagnostic medicine, and its critical usefulness was found in the COVID-19 pandemic [32, 33]. It has also played a critical role in genome sequencing, drug, and vaccine formulation, preparing for outbreaks, monitoring disease distribution, and logging virus variants [34]. Automated reasoning systems are used to detect disease outbreaks early and track worldwide illness incidence in significant amounts of datasets [19]. The learning-based approach has boosted attention by permitting various learning strategies to be integrated into these models and offering far more significant datasets, such as clinical and sociodemographic variables, to be added [35]. The method of computational incorporation requires enhancing surveillance and screening capacities in areas with limited resources. The use of AI in infectious disease systems complements traditional approaches and scales massive study and sampling metrics [33]. AI greatly augments infectious disease systems as it can provide complementarity to traditional modalities with massive study and sampling metrics. AI algorithms can detect an epidemic much faster than traditional methods [36]. AI-based systems also detected outbreaks up to 10 days ahead of conventional means during the COVID-19 pandemic. Moreover, AI-based systems are capable of monitoring and analyzing lots of data in great detail from multiple resources or information feeds like social media platforms, climatic data, and epidemiological records that allow real-time tracking and instant detection of infection patterns [37]. The rapid processing of data enables timely detection and prediction of outbreaks, leading to timely interventions and thus improving public health outcomes [38].  Table 1 demonstrates how AI has been used for early infectious disease detection and diagnosis; nevertheless, using AI without precautions also has its disadvantages and constraints [32, 48].

However, employing it still takes expert advice [34]. Alternatively, the expenses or time needed for standard diagnostic methods, such as microscopy or swift diagnostic test kits, are limiting. The outcomes of these methods were unsatisfying [49]. AI-based systems could tackle these issues, allowing for quick, powerful detection without professional decision making as deep learning techniques and image appearance help achieve improved accuracy in quickly assessing infectious diseases. There are challenges and constraints; however, AI-aided simulations display promising results.

### 2.3. Challenges and Limitations

Compared to the existing practices, AI models have the potential to improve the diagnosis accuracy and precision of the results. Thanks to ML approaches, AI models continue to learn from new data exposure, making it possible to adjust their diagnosis patterns and continually improve them. Both accuracy and precision are crucial for the diagnosis and prognosis of infectious diseases. “Advancements in biomedical technologies and the availability of large clinical and omic data and the proper use of AI promote precision medicine in the area of infectious diseases and vaccines” [50]. While precision medicine is not yet widespread in the case of infectious diseases, it can help “to identify the determinants of adverse and protective clinical outcomes and inform targeted approaches for optimal patient management, personalization of treatment strategies, and vaccination” [51]. Precision epidemiology also enhances the responses to infectious disease outbreaks with the possible personalization of the diagnosis, tracking, and treatment [52]. In healthcare, accurate diagnosis ensures that clinicians can develop nursing interventions that target the needs of the specific population, which further improves patient outcomes [53]. Accurate and precise diagnosis and prognosis are fundamental for the effective prevention and management of infectious diseases. Despite the potential benefits, accurate and precise diagnosis and prognosis face several challenges. Some pathogens are rare and unknown, or the microbes causing the infection may behave differently because of gene changes [54, 55]. Some of them are underdiagnosed and misdiagnosed because they are not frequent. Regular culture-based techniques cannot detect polymicrobial and fungal infection cases, which are not easily culturable [56]. The challenge of the lack of diagnostic test accuracy forms and standards also complicates the picture, particularly when more effective tests replace less reliable existing tests [57]. Considering that many clinical conditions present similarly for viral and bacterial infections, such as pharyngitis, otitis media, and acute respiratory illness, accurate diagnosis is difficult with the currently available methods [58]. An elevated level of diagnostic uncertainty also characterizes several infectious diseases. Clinical uncertainty reaches high levels across several clinical conditions, complicating clinical management and antibiotic optimization [59].

## 3. Prognosis of Infectious Diseases

**Figure 1:**
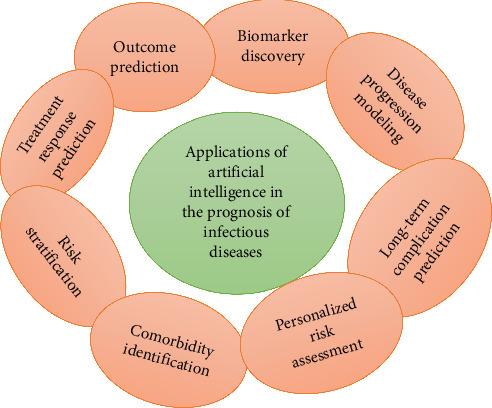
Illustration of various applications of AI in the prognosis of infectious diseases.

A brief overview of what AI-driven prognoses look like for their applications in infectious disease diagnosis is illustrated in Figure 1, distinguishing important applications including outcome prediction, biomarker discovery, longitudinal complication prediction, patient-specific risk assessment, comorbidity identification, and treatment response prediction, to present complex elements in easier parts to analyze visually. It emphasizes the essence of using AI to improve the accuracy and efficiency of disease prognosis in predictive health. Presenting the applications visually is a smart way to display otherwise sordid topics even for healthcare professionals and patients, who may not understand the full potential of AI in managing infectious diseases. AI algorithms analyze patient data in all fields, including demographics, clinical symptoms, laboratory results, and medical history. More specifically, AI models have the potential to predict how patients with infectious diseases may respond to specific treatments, such as antibiotics or antiviral medications [34, 60].

AI may assist healthcare providers in planning resources and developing individualized treatment strategies by segregating patients based on their risk of complications or severe illness [61]. It also identifies any pre-existing illness or risk factors that might increase the severity of infectious diseases or influence the results of treatment possible, making it easier for clinicians to provide patient-centered care [62]. Alternatively, AI may investigate biological data to identify new biomarkers for infectious diseases. This involves studying gene expression patterns, protein concentrations, and metabolic processes that are vital for prognosis and treatment selection [63]. Similarly, AI models simulate the progression of infectious diseases in people or populations, taking into account pathogen defenses, host biology, and environmental aspects [64, 65]. Such a model helps predict patients' prognosis and design public health policy. AI can also forecast the possibility of chronic illnesses or sequela after an infectious disease, such as chronic seasonal flu. This information is ideal for proper disease management and is critical for further follow-up. Finally, AI combines individual patient data and population statistics with network knowledge and ML methods to generate individual risk scores. By doing so, AI will enhance the accuracy of prognoses and support a patient-centered approach, enabling individual risk assessment to guide clinical judgment and advance personalized medicine [66]. ML models more accurately predicted and assessed clinical diagnosis, therapeutic efficacy, epidemic progression, and diagnosis speed based on the patient's clinical characteristics, laboratory tests, and imaging examination [34]. AI was used to locate severe disease outbreaks or predict them, primarily prevalent blood-feeding arthropod, vector-borne diseases [67]. Traditional methods to validate vector populations are time-consuming, yet AI, notably deep learning, has been utilized to categorize mosquito species based on acoustic signaling [68]. However, there are still some challenges, such as diagnostic and therapeutic discomfort. Clinicians may make errors due to diagnosis and treatment difficulties, Clinicians may make errors due to challenges in diagnosis and treatment, resulting in misdiagnosis, inappropriate diagnoses in many cases, and overtreatment [69]. The future of AI in infectious disease outbreaks and pandemic control is predicted as auspicious [70].

## 4. Personalized Diagnosis and Prognosis

AI analyzes patient data comorbidities, lifestyle factors, and genomic sequencing to propose personalized treatments. AI can use comorbidities to find potential interactions among various health problems and adapt treatment plans for that complexity. AI algorithms also consider lifestyle factors, like diet, exercise, and smoking habits, to suggest interventions best suited to the patient's daily routines and habits. Genomic sequencing gives a comprehensive profile of genetic information from the patients themselves, enabling AI to target specific genetic mutations or variations that could impact the efficiency of treatments [71, 72]. AI can then utilize these various data types, combined, to provide long, personalized, and meaningful treatment recommendations that will not only enhance the individual but also ultimately alleviate the strain on the healthcare system. Furthermore, AI-driven diagnostic and prognostic models ensure individualized treatment strategies that match patients and their specific affected characteristics. AI has made a significant impact on personalized diagnosis and prognosis in infectious diseases [32, 73]. AI has progressively enhanced the accuracy and efficiency of diagnostic predictions within the domain of infectious diseases. For instance, in the field of epidemic detection, AI algorithms have shown they can detect epidemics 10 days sooner than traditional surveillance methods [74]. Moreover, AI-powered algorithms possess the ability to immerse themselves in an aggregate of open data from social media, climate-related reports, and epidemiology, facilitating real-time surveillance and the swift detection of infection patterns. ML algorithms have improved disease typing and diagnosis by assessing clinical images, lab results, and patient data, providing timely and accurate diagnoses [73]. However, these improvements have also resulted in optimized resource allocation, timely interventions, and better public health outcomes [75].

AI-driven approaches to diagnosis and prognosis include diagnostic analysis, drug and vaccine development, identification of outbursts and patients, tracking of outbreaks, identification of new strains, and other aspects of medicine, including genetics [68, 76]. AI has the potential to identify complex patterns in large datasets, aid in medical decision making, diagnose diseases more quickly, match treatment types, and reduce mistakes [77]. ML modeling has proven to be highly effective, precise, and understandable in developing an infectious disease model using patient clinical characteristics, laboratory measurements, and imaging studies [78]. Clinical microbiology has evolved from manual suboptimal, labor-intensive, and time-consuming detection and identification procedures to the use of automation and AI in diagnosis [79]. Thus, AI in infectious disease diagnosis has both its challenges and avenues for success [68]. AI-based predictive models can be utilized to assess and enhance clinical disease diagnosis, detect epidemics, and evaluate the therapeutic impact of treatments and patient education. These applications can lead to both positive and adverse outcomes for patients [34, 80]. Other challenges include the quality of clinical data used by AI models and social and human factors associated with the introduction of AI in infectious diseases [81]. Future possibilities promise many benefits: AI is likely to deepen our understanding of infection biology, increase the accuracy of infection diagnostics, and minimize the threat of large-scale outbreaks. AI and ML systems can detect important patterns of infection and disease in patients at an early stage and decrease infection risk [82]. For patient care, the next steps should include building predictive analytics in point-of-care testing and integrating AI and ML into data fusion applications.

## 5. Implications of AI for Healthcare Delivery

AI integration into infectious disease diagnostics and prognostics offers immense possibilities to advance healthcare outcomes and strengthen pandemic preparedness; however, it is critical to address the limitations discussed above to realize these possibilities. While ensuring high-quality and transparent data and considering ethical aspects during AI deployment, other critical dimensions include advocating for enhancing regulatory frameworks, supporting infrastructure, and training the workforce. The essential collaborative effort between healthcare providers, researchers, policymakers, and technology developers will be necessary to overcome implementation hurdles and enable the benefits of AI for infectious disease management [34]. Its implications for healthcare delivery in infectious diseases include anti-infective drug discovery, infection biology understanding, diagnosis, infection presence and propagation detection, and vaccine efficacy enhancement [83]. AI integration in surgical site infections aids in prompt wound colonization detection and bacteria breathing, resulting in immediate antibiotic administration and vast patient outcome improvement. The recent application worth noting includes Chatbot Generative Pretrained Transformer which has opened new possibilities to clinical practice and scientific research in infectious diseases [81]. AI applications in healthcare solve different problems, including medical imaging and diagnostics, virtual patient consultation, medical research and drug discovery, patient engagement and follow-up, rehabilitation, and administrative work, mainly for meeting the healthcare needs of tomorrow [84]. Although AI in healthcare has enormous potential to benefit future healthcare, there are numerous technical, ethical, and social challenges to ensuring patient safety, accountability, and patient and caregiver acceptance [85].

## 6. Data Availability, Quality, and Security

The diagnosis and prognosis of infectious diseases face challenges due to the availability, quality, and security of data. During pandemics, the scientific community and governments in almost every country in the world struggle to obtain up-to-date data and evidence for informed decision making, which hinders effective responses [86]. However, all of these high-quality biomedical datasets are essential for infectious disease research and are increasingly facing fairness challenges [87]. The lack of metadata standards in repositories hinders both findability and accessibility, thereby hindering data discovery and reuse. Furthermore, while clinical decision support systems (CDSSs) have significant potential to improve healthcare management of infectious diseases, their implementation in real-world applications is still limited, necessitating their evaluation and integration into daily clinical workflows for increased effectiveness [88]. This becomes even more crucial in resource-constrained countries like all those in Africa, necessitating the development of bespoke multiplex point-of-care diagnostic tools to effectively address the challenges of acute infection diagnosis and control [89]. Advances in technology and data analytics have significantly influenced the availability, quality, and security of data used for identifying and understanding infectious diseases. More recently, researchers have started analyzing disease outbreaks and enhancing disease surveillance using ML algorithms, big data analysis, and AI [90]. These technologies enhance the accuracy of forecasts by integrating information from various sources, such as medical records. However, the use of incomplete or poorly informative medical data may compromise the reliability of disease outbreak predictions [91]. New data sources, such as those based on technology-enabled physiological measurements and AI, are being considered to strengthen infectious disease surveillance by allowing faster, more resource-efficient approaches compared to traditional methods [92].

On the other hand, the creation of novel health data analysis techniques supported by human behavior data has opened new pathways to fight infectious diseases, as it stands out during the current COVID-19 pandemic [93]. Various risks and ethical considerations are associated with the use of sensitive patient data in infectious disease diagnosis and prognosis. The balance between protecting patient privacy and maintaining data integrity for predictive analytics is one of the key considerations here [94]. Sharing EHRs for research is fraught with ethical dilemmas related to informed consent, data quality, and confidentiality, underscoring the requirement for good judgment [95]. Furthermore, the involvement of private, for-profit enterprises in the search for patient databases raises additional data protection concerns, potentially leading to the use and sale of patient data for commercial purposes and targeting vulnerable groups. This underscores the significance of patient data–sharing governance and the ethical foundations of data use in research [96]. Moreover, in medical research, the disclosure of too many patient traits may lead to the loss of anonymity and confidentiality for the patient, and therefore, one must limit the information disclosed about a given attribute to protect patient information [97].

## 7. Implementation of AI Models

AI models are essential for the diagnosis and prognosis of infectious diseases because they can integrate clinical features, laboratory tests, and imaging measures to predict clinical manifestation, treatment response, and outbreak alert [68]. Logistic regression, support vector machines (SVMs), decision trees, and ensemble techniques are models that could improve the early detection and management of diseases such as TB, influenza, HIV, and COVID-19 [32, 98]. Specific challenges are addressed using distinct advantages offered by common AI models used in infectious disease prognosis. Logistic regression is primarily used to establish the probability of binary responses such as whether the disease occurs or not (1 or 0) at a time and works well with small datasets with a linear relationship [99]. SVMs perform well in high-dimensional spaces and will be beneficial for nonlinear data and therefore could rapidly classify patients based on clinical and genomic data [100]. Decision trees ease the process of making decisions with a set of rules that can be quickly visualized and require minimal preprocessing, making them valuable for identifying infection risk factors [101]. Ensemble methods like random forests and gradient boosting improve the predictive accuracy and robustness of models by combining multiple approaches which makes them suitable for predicting disease outbreaks and discovering comorbidities [102]. Both models bring their unique strengths to the table, addressing the multifaceted complexities of infectious disease management.

Data handling limitations relate to data types, quality, and availability, necessitating the use of diverse and high-quality data for accurate modeling [103]. Secondly, networks and international cooperation should implement AI-based strategies for infectious disease surveillance and pandemic preparedness, enabling the use of AI in future pandemic surveillance programs [67]. AI models dramatically improve the diagnosis and prognosis of infectious diseases by discovering patterns and forecasting results through complex analysis of substantial amounts of medical data. An application is using ML algorithms to analyze CT scans to diagnose COVID-19-related pneumonia more quickly and often more accurately than human radiologists [104]. In the end, these models have the potential to forecast disease progression, leading to more efficient allocation of medical resources. However, the problem is that they require high-quality, diverse datasets that are accurately trained to be effective, and if the data are not truly indicative of the class, it can develop undesirable biases. Moreover, the interpretability of AI models themselves can be a challenge, which means that healthcare providers may fail to fully trust and understand the recommendations given by an AI model.

## 8. Human Roles With AI-Driven Algorithms

With such AI-driven algorithms, human roles are changing in the diagnosis and prognosis of infectious diseases. Researchers have proven these ML models to be efficient, accurate, and easily interpretable, making them helpful and easy to use in clinical patient or laboratory measurement and image screening for early detection and prediction of outbreaks [32]. Such AI algorithms enhance the customary public health surveillance for early containment of infectious diseases such as TB, influenza, HIV, and COVID-19 [32]. The increase in MDR microorganisms triggering infections is growing worldwide and becoming more serious in developing countries [17]. In recent years, the infectious diseases due to the emergence of MDR bacteria have been increased [105, 106]. Combining human experts with AI has made it easier to analyze Gram stain images, read culture plates automatically, and find pathogens in clinical microbiology [68]. This has led to better infection control, prevention, and epidemiology. As AI development progresses, human input is critical in interpreting AI insights, verifying data quality, and directing the application of AI-enabled tools for infectious disease surveillance and pandemic preparedness [73, 103]. Medical professionals, including doctors and radiologists, also utilize AI to enhance their decision-making abilities. A radiologist could quickly verify this disease while decreasing diagnostic errors by using an AI algorithm to review chest X-rays and identify TB [107]. The clinician is responsible for making the ultimate diagnosis and treatment determination, combining AI insights with their own clinical experience and patient-specific information. This symbiosis ensures that human judgment can leverage AI data processing for the mutual enrichment of healthcare or clinical decisions. AI indeed has many limitations, but still, it is up to the healthcare providers to know the extent to which they can rely on AI or what checks they should be doing to validate AI outputs.

## 9. Recent Developments

AI-driven algorithms enhance earlier and more accurate diagnoses of infectious disease [108, 109]. These algorithms can digest substantial amounts of data such as genomic sequences and medical images. Using advances in deep learning models to identify patterns even fetoscopy ones, these healthcare technologies can diagnose diseases such as pneumonia and TB more rapidly using medical images [110, 111]. With predictive modeling, AI is improving ML models to predict disease outbreaks based on environmental, social, and epidemiological data [112]. AI is a true asset in the fight against infectious diseases as it not only enhances diagnostic accuracy but also makes treatment more efficient and personalized. The innovative prospect of transforming infectious disease diagnosis and prognosis with the current AI technologies under a broader context is our interest in this paper. The mixing of AI with genomic sequencing has helped in revolutionizing what pathogens have caused infections or deaths and bringing about measures to help control emerging infectious ailments which were observed during COVID-19 [113]. Genomic sequencing technology combined with AI assists in transforming the way microorganisms are detected and controlled. By comparing genomic sequences with known databases, AI algorithms can quickly and accurately identify pathogens [114]. These models can be used to analyze genomic data in real time (for example, to monitor outbreaks and genetic variation and track the spread of infectious diseases) to inform interventions [115]. AI also identifies genetic signatures related to AMR, aiding in the formulation of custom treatments [116]. AI also guides public health, predicting how infectious disease outbreaks will spread and affect populations by combining genomic data with environmental, social, and epidemiological signals [117]. It also aids in personalizing treatments based on an individual's genetic composition, suggesting customized therapies for improved results [118]. These tools, when combined with genomic sequencing, improve pathogen prediction, outbreak response, and novel treatment discovery. Clinical data for predicting the prognosis of COVID-19 patients demonstrate that AI-tailored care is significantly more effective in identifying poor outcomes and risk factors. Such developments are facilitating quicker and more personalized healthcare solutions that can result in improved patient outcomes while reducing public health threats [119].

Similarly, federated learning is a ML technique, where multiple institutions can jointly train a ML model without sharing raw data themselves [120]. This becomes especially handy in the healthcare domain, where patient data confidentiality is crucial. This means that federated learning maintains data security and compliance with regulations as the data do not leave its local environment, and only model updates are shared. In contrast, blockchain technology presents a decentralized and secure method for handling healthcare data [121]. Its ability to improve data integrity, transparency, and security makes it well-suited for applications like EHRs, supply chain management, and even patient consent management [121]. This immutability feature of blockchain offers patients' healthcare data a productive and accurate way to hold it on the ledgers and not allow any alterations, ensuring that whatever is provided is true and reliable information. Both technologies tackle some of the biggest points in healthcare like data privacy, security, and interoperability [121]. They also set the stage for innovative and patient-centric healthcare solutions.

## 10. Ethical Considerations

Despite the possible implications, the application of AI in the diagnosis and prognosis of infectious diseases in a patient raises serious ethical concerns, including but not limited to patient privacy, the possible bias of the AI algorithm, and equity and access to healthcare. These concerns need to be addressed to deploy AI for such applications responsibly and equitably. The various ethical considerations in the application of AI to enhance the early detection and diagnosis of infectious diseases include patient privacy, data integrity, bias, liability, and transparency. These concerns need to be addressed for the responsible use of AI in healthcare [122]. Several other dimensions of ethical issues go along with the use of AI in healthcare and other sectors, such as respect for people, liability, and sustainability. The abovementioned ethical concerns need to be resolved through a deliberative democratic process and the creation of a societal consensus [123]. The Common Rule principles in AI applications in public health encompass equity, privacy, security, safety, transparency, confidentiality, liability, social equity, and autonomy. These principles need to be developed into guidelines to make AI deployment in public health responsible [124]. In the context of the use of AI in the speech-based diagnosis of Alzheimer's disease, concerns about autonomy, privacy and the security of data, welfare and the protection of human rights, transparency, and fairness apply. These concerns should be formulated in a set of guidelines for researchers and clinicians to follow [125]. The use of AI in the prediction and personalized diagnosis and prognosis of infectious diseases also has ethical implications. The implementation of ethical frameworks when using AI that support decision making can solve the problem of antimicrobial overuse and resistance [123, 126]. The advancements in AI have significantly improved access to healthcare, diagnosis, medicine creation, and disease prevention. Nonetheless, while AI has helped reduce the burden of diagnosis and treatment, surveillance and prediction ability, and medicine and vaccine development efficiency, low-effort diagnosis, lack of open space–based disease surveillance and prediction, and weak intellectual system service delivery are some of its limitations [127]. Therefore, improving multimodal diagnostic systems, focusing on open environment surveillance and prediction, and increasing ethical and big data–based security can help solve this problem [40].

## 11. Barriers to Practical Implementation

The use of AI technology in healthcare can be very costly and can be almost beyond the reach of low-income countries. The cost of AI integration includes capital expenses, which cover the purchase of hardware, software, and other components. AI systems require ongoing maintenance and periodic upgrades to sustain and modernize them, incurring continuing costs. Moreover, healthcare professionals must be trained on how to use these AI tools effectively, which requires both time and financial resources. The expenses of collecting, storing, and managing the massive datasets needed for these algorithms can also be hefty, and ensuring data privacy and security only increases [128]. In low- and middle-income countries, there are significant challenges for AI applications due to a lack of infrastructure [129]. In these areas, many healthcare facilities do not possess the advanced technologies and functional Internet connectivity required by AI systems. Integrating AI tools into an existing healthcare infrastructure is impossible in some parts of the world where there are no diagnostic machines or EHR systems already in place. AI systems are dependent on stable power, and an unreliable electricity supply could hamper their operation. Similarly, AI systems require extensive and high-quality datasets, but data collection can be fragmented, incomplete, or poor quality within many low-income countries making AI algorithms less effective.

The workforce is equipped to implement AI successfully in the healthcare sector. However, there is usually a lack of healthcare professionals trained in AI and data science skills in low-income nations. In the knowledge economy, this migration fills the gap for qualified professionals but reduces the number of qualified personnel in the home country. Healthcare workers may also be resistant to adopting new technologies due to a lack of familiarity or fear of displacement [130]. Barriers to AI implementation are also caused by ethical and regulatory challenges. Protecting the privacy and security of patient data is essential yet difficult, particularly in areas with lax regulatory regimes. If improperly designed and overseen, AI algorithms can carry over pre-existing biases from healthcare, resulting in disparities in the availability of care and treatment. Regulatory approval of AI tools can be a slow, complex process, especially in countries with poorly developed regulatory systems [131]. Some strategies to address these barriers include knowledge transfer, resource sharing, and capacity building. Many of these are already in place, as countries can form partnerships to establish capacity-building initiatives, bridges, and knowledge-sharing models [132]. By implementing strong ethical and regulatory frameworks, we can ensure the responsible and equitable use of AI in healthcare.

## 12. Strengths and Limitations

AI algorithms can analyze diverse datasets to identify subtle patterns indicating infectious diseases, allowing for earlier detection compared to traditional methods [133]. This early detection is crucial for timely intervention and containment efforts, reducing the spread of the disease. AI-driven models can consider individual patient characteristics to customize treatment strategies, optimizing effectiveness and minimizing adverse effects. This personalized approach has the potential to improve patient outcomes and reduce healthcare costs. AI enables real-time analysis of epidemiological data, enabling proactive measures to control disease outbreaks [134]. Predictive modeling capabilities empower public health authorities to effectively allocate resources and implement targeted interventions. AI accelerates the analysis of large-scale genomic and proteomic data, facilitating the identification of new pathogens and therapeutic targets [135, 136]. This has implications for vaccine development, drug discovery, and precision medicine approaches.

However, for AI models to make accurate predictions, they rely on high-quality and representative data. Biases in training datasets can lead to algorithmic bias and inaccurate results, particularly in underrepresented populations or regions [137]. The opaque nature of some AI algorithms makes interpretation and transparency difficult, raising concerns about the reliability of diagnostic and prognostic outputs [138, 139]. Clinicians may be hesitant to rely on AI-driven recommendations without understanding the underlying rationale. Regulatory frameworks are often slow to catch up with technological advancements, posing challenges in ensuring responsible AI deployment and adherence to ethical standards. Healthcare systems also face practical challenges in integrating AI technologies into existing workflows. Technical infrastructure requirements, workforce training needs, and financial investments may hinder the widespread adoption of AI-driven diagnostic and prognostic models.

## 13. Opinion and Future Perspective

Infectious diseases pose a significant global health challenge, requiring innovative approaches to timely diagnosis, prognosis, and management. The integration of AI into diagnostic and prognostic models for infectious diseases is a groundbreaking advancement that has the potential to revolutionize healthcare. Expert opinions on this prospect emphasize both the immense promise and critical challenges associated with using AI in infectious disease management. AI algorithms will continue to improve their ability to analyze large amounts of data from various sources, enabling earlier and more accurate detection of infectious diseases. This early detection will facilitate swift public health responses, including containment and targeted interventions, reducing the spread of infections, and minimizing their impact on populations. As AI models become more sophisticated, they will consider individual patient characteristics, such as genetics, comorbidities, and lifestyle factors, to tailor treatment strategies for infectious diseases. This personalized approach will optimize treatment efficacy, reduce adverse effects, and improve patient outcomes. AI-enabled predictive models will evolve, providing real-time insights into disease trends and accurately forecasting outbreaks. By analyzing diverse data sources, including social media, Internet searches, and wearable device data, these models will empower public health authorities to implement initiative-taking measures to contain infectious diseases before they escalate into epidemics or pandemics. AI technologies will play a crucial role in rapidly identifying and characterizing emerging infectious disease threats, such as new pathogens or antimicrobial-resistant strains. By using ML algorithms to analyze genomic sequences and other data, researchers and public health experts will be better equipped to develop targeted interventions, including vaccines and therapeutics, to mitigate the spread of emerging infectious diseases.

The integration of AI into telemedicine platforms and remote monitoring devices will enable more efficient and accurate diagnosis and management of infectious diseases, particularly in remote or underserved areas. AI-powered chatbots and virtual assistants will provide personalized guidance and support to patients, helping them navigate their illnesses and adhere to treatment protocols. Regulatory frameworks need to evolve to ensure the responsible and equitable use of AI-driven diagnostic and prognostic models while safeguarding patient rights and privacy. Collaboration among healthcare providers, researchers, policymakers, and technology developers is essential for advancing the field of AI-driven infectious disease diagnostics and prognostics. Global knowledge sharing and collaboration will accelerate innovation, facilitate the development of best practices, and ensure that AI technologies benefit all populations, regardless of location or socioeconomic status. While the future of integrating AI diagnostic and prognostic models for infectious diseases is promising, ongoing attention to ethical, regulatory, and equity considerations is crucial to realizing AI's full potential in combating infectious diseases and promoting global health and well-being. It is important to remember that healthcare is primarily a human endeavor, and as AI becomes more integrated into healthcare, we must evaluate its impact on the fundamental human connections that underpin healthcare practice. While AI offers unprecedented opportunities to improve healthcare outcomes and enhance public health surveillance, we must address critical challenges to ensure responsible use, ethical considerations, and equitable access to AI-enabled healthcare solutions. Collaborative efforts among stakeholders are essential to realizing the promise of AI in combating infectious diseases and advancing global health equity. As we navigate the complexity of integrating AI into healthcare, we must prioritize patient safety, privacy, and equity while harnessing AI's transformative power to create a healthier world for all.

## 14. Conclusion

In conclusion, this review highlights the transformative potential of AI in diagnosing and predicting the progression of infectious diseases. The introduction of AI technologies has ushered in a new era for healthcare, with its potential to revolutionize various aspects and significantly drive forward innovations in early detection, personalized treatment, and efficient public health system management, ensuring better patient outcomes and public health initiatives. Nonetheless, leading the successful integration of AI with healthcare systems needs to address many challenges ranging from data privacy and algorithm bias to ethical considerations. This review also offers directions for potential solutions by recognizing these challenges and therefore writes a new chapter of insights for researchers, healthcare professionals, and policymakers. The future perspective in this key research topic area is to continue to address these barriers and to evolve AI technologies to further derive the promise of infectious disease management. These insights and recommendations will play an important role in achieving the full potential of AI to transform healthcare delivery and equity of access to its benefits. In addition, AI technology should collectively study with other technologies (e.g., blockchain and the Internet of Things (IoT)) to improve data security and cooperation in medical care systems. Given these factors, the future of AI in healthcare will necessitate not only advancements in technology but also a strong focus on interdisciplinary collaborations to address the ethical and social implications of AI in healthcare, thereby ensuring that its benefits are equitably distributed across diverse populations.

## Figures and Tables

**Table 1:** Outlining some ways AI is used in the diagnosis of infectious diseases.

Application	Description	Reference
Detection of pathogens	AI algorithms can analyze biological samples, such as blood or tissue, to identify the presence of pathogens such as bacteria, viruses, or fungi	[39]
Predictive modeling	AI can use data from climate patterns, population movements, and historical infection rates to predict disease outbreaks. These predictive models assist healthcare providers in allocating resources and implementing preventive measures	[40]
Drug discovery	AI speeds up the drug discovery process by analyzing extensive datasets to identify potential drug candidates. It can predict the effectiveness and safety of compounds, thereby expediting the development of treatments for infectious diseases	[41, 42]
Diagnostic imaging analysis	AI algorithms are used to analyze medical images, including X-rays, CT scans, and MRIs. Their purpose is to detect signs of infectious diseases like pneumonia or tuberculosis. By doing so, these tools help radiologists with precise diagnosis and treatment planning	[43, 44]
Epidemiological surveillance	AI monitors online sources, social media, and news reports to track the real-time spread of infectious diseases. Through the analysis of big data, AI assists public health authorities in promptly responding to outbreaks and implementing precise interventions	[19, 45]
Antibiotic resistance prediction	AI algorithms analyze genetic data from pathogens to predict their resistance to antibiotics. This valuable information assists clinicians in choosing the most effective treatment for infectious diseases and plays a crucial role in the fight against antibiotic resistance	[46]
Remote monitoring	AI-powered wearable devices and mobile apps enable the remote monitoring of vital signs and symptoms related to infectious diseases. This allows patients to receive timely interventions, improves patient outcomes, and lowers healthcare costs	[47]

## Data Availability

All data are provided with references in the text and the reference list.

## References

[1] Wang Y., Fu E. Y., Zhai X., Yang C., Pei F. (2024). Introduction of Artificial Intelligence. *Intelligent Building Fire Safety and Smart Firefighting*.

[2] Nyholm S. (2024). Artificial Intelligence and Human Enhancement: Can AI Technologies Make Us More (Artificially) Intelligent?. *Cambridge Quarterly of Healthcare Ethics*.

[3] Cui Y., Hu W., Rahmani A. (2024). Multi-Robot Path Planning Using Learning-Based Artificial Bee Colony Algorithm. *Engineering Applications of Artificial Intelligence*.

[4] Pongtambing Y. S., Appa F. E., Siddik A. M. A. (2023). Peluang Dan Tantangan Kecerdasan Buatan Bagi Generasi Muda. *Bakti Sekawan: Jurnal Pengabdian Masyarakat*.

[5] Alnaji L. (2024). Machine Learning in Epidemiology: Neural Networks Forecasting of Monkeypox Cases. *PLoS One*.

[6] Kumar V., Iqbal M. I., Rathore R. (2025). Natural Language Processing (NLP) in Disease Detection—A Discussion of How NLP Techniques Can Be Used to Analyze and Classify Medical Text Data for Disease Diagnosis. *AI in Disease Detection: Advancements and Applications*.

[7] Beri M., Gill K. S., Upadhyay D., Devliyal S. AI-powered Pneumonia Detection: Enhanced Chest X-Ray Interpretation with CNNs.

[8] Aljaaf A. J., Ibrahim M., Al-Ouqaili M. T. N. Perspectives on Industry 4.0 Awareness Among Undergraduate IT Students in IRAQ: University of Anbar as a Case Study.

[9] Dwivedi Y. K., Hughes L., Ismagilova E. (2021). Artificial Intelligence (AI): Multidisciplinary Perspectives on Emerging Challenges, Opportunities, and Agenda for Research, Practice and Policy. *International Journal of Information Management*.

[10] Beam A. L., Drazen J. M., Kohane I. S., Leong T.-Y., Manrai A. K., Rubin E. J. (2023). Artificial Intelligence in Medicine. *Massachusetts Medical Society*.

[11] Nabiyeva F., Umarova S., Umirkulova S. (2023). Artificial Intelligence in Medicine. *Journal of New Century Innovations*.

[12] Akyon S. H., Akyon F. C., Yılmaz T. E. (2023). Artificial Intelligence-Supported Web Application Design and Development for Reducing Polypharmacy Side Effects and Supporting Rational Drug Use in Geriatric Patients. *Frontiers of Medicine*.

[13] Amiri Z., Heidari A., Darbandi M. (2023). The Personal Health Applications of Machine Learning Techniques in the Internet of Behaviors. *Sustainability*.

[14] Rajput H., Tiwari S. K. (2023). Detection of Diseases and Predictive Analytic. *Detection Of Diseases And Predictive Analytic*.

[15] Khalate P., Gite S., Pradhan B., Lee C.-W. (2024). Advancements and Gaps in Natural Language Processing and Machine Learning Applications in Healthcare: A Comprehensive Review of Electronic Medical Records and Medical Imaging. *Frontiers in Physics*.

[16] Gao A., Murphy R., Chen W. (2021). Progress in Robotics for Combating Infectious Diseases. *Science Robotics*.

[17] Farajzadeh Sheikh A., Moradi Bandbal M., Saki M. (2020). Emergence of Multidrug-Resistant Shigella Species Harboring Extended-Spectrum Beta-Lactamase Genes in Pediatric Patients With Diarrhea from Southwest of Iran. *Molecular Biology Reports*.

[18] Msemburi W., Karlinsky A., Knutson V., Aleshin-Guendel S., Chatterji S., Wakefield J. (2023). The WHO Estimates of Excess Mortality Associated With the COVID-19 Pandemic. *Nature*.

[19] MacIntyre C. R., Chen X., Kunasekaran M. (2023). Artificial Intelligence in Public Health: The Potential of Epidemic Early Warning Systems. *Journal of International Medical Research*.

[20] Garbacz K., Wierzbowska M., Kwapisz E. (2021). Distribution and Antibiotic-Resistance of Different Staphylococcus Species Identified by Matrix Assisted Laser Desorption Ionization-Time of Flight Mass Spectrometry (MALDI-TOF MS) Isolated From the Oral Cavity. *Journal of Oral Microbiology*.

[21] Saki M., Sheikh A., Seyed-Mohammadi S. (2022). Publisher Correction: Occurrence of Plasmid-Mediated Quinolone Resistance Genes in *Pseudomonas aeruginosa* Strains Isolated from Clinical Specimens in Southwest Iran: A Multicentral Study. *Scientific Reports*.

[22] Chouit E. M., Rachdi M., Bellafkih M., Raouyane B. (2024). Harnessing the Power of Artificial Intelligence and the Internet of Things for Improved Epidemic Forecasting. *Advancements in Climate and Smart Environment Technology*.

[23] Isiaka A., Anakwenze V., Ilodinso C. (2024). Harnessing Artificial Intelligence for Early Detection and Management of Infectious Disease Outbreaks. *International Journal of Innovative Research and Development*.

[24] World Health Organization (2011). World Malaria Report 2011. http://www.who.int/malaria/world_malaria_report_2011/en/index.html.

[25] Tang K., Millar B., Moore J. (2023). Antimicrobial Resistance (AMR). *British Journal of Biomedical Science*.

[26] Palakurti N. R. (2025). Obstacles and Resolutions: Integrating Artificial Intelligence in Sustainable Development. *Artificial Intelligence and Machine Learning for Sustainable Development*.

[27] Ankolekar A., Eppings L., Bottari F. (2024). Using Artificial Intelligence and Predictive Modelling to Enable Learning Healthcare Systems (LHS) for Pandemic Preparedness. *Computational and Structural Biotechnology Journal*.

[28] Srivastava V., Kumar R., Wani M. Y., Robinson K., Ahmad A. (2025). Role of Artificial Intelligence in Early Diagnosis and Treatment of Infectious Diseases. *Infectious Diseases*.

[29] Li J., Tian Y., Zhou T. (2024). Electronic Medical Record Systems. *Healthcare Information Systems: Progress, Challenges and Future Directions*.

[30] Gupta Y. D., Bhandary S. (2024). Artificial Intelligence for Understanding Mechanisms of Antimicrobial Resistance and Antimicrobial Discovery: A New Age Model for Translational Research. *Artificial Intelligence and Machine Learning in Drug Design and Development*.

[31] Hutchinson D., Kunasekaran M., Quigley A., Moa A., MacIntyre C. (2023). Could It Be Monkeypox? Use of an AI-Based Epidemic Early Warning System to Monitor Rash and Fever Illness. *Public Health*.

[32] Parums D. V. (2023). Infectious Disease Surveillance Using Artificial Intelligence (AI) and Its Role in Epidemic and Pandemic Preparedness. *Medical Science Monitor: International Medical Journal of Experimental and Clinical Research*.

[33] Sisimayi C., Harley C., Nyabadza F., Visaya M. V. (2023). AI-Enabled Case Detection Model for Infectious Disease Outbreaks in Resource-Limited Settings. *Frontiers in Applied Mathematics and Statistics*.

[34] Wong F., de la Fuente-Nunez C., Collins J. J. (2023). Leveraging Artificial Intelligence in the Fight Against Infectious Diseases. *Science*.

[35] Alqaysi M., Albahri A., Hamid R. (2022). Hybrid Diagnosis Models for Autism Patients Based on Medical and Sociodemographic Features Using Machine Learning and Multicriteria Decision-Making (MCDM) Techniques: An Evaluation and Benchmarking Framework. *Computational and Mathematical Methods in Medicine*.

[36] Zhao A. P., Li S., Cao Z. (2024). AI for Science: Predicting Infectious Diseases. *Journal of Safety Science and Resilience*.

[37] Non L. R., Marra A. R., Ince D. (2025). Rise of the Machines-Artificial Intelligence in Healthcare Epidemiology. *Current Infectious Disease Reports*.

[38] Hsu J., Lu C., Hsu M. (2024). Artificial Intelligence in Infectious Diseases: Pathogenesis and Therapy. *Frontiers of Medicine*.

[39] Horta-Velázquez A., Arce F., Rodríguez-Sevilla E., Morales-Narváez E. (2023). Toward Smart Diagnostics via Artificial Intelligence-Assisted Surface-Enhanced Raman Spectroscopy. *TrAC, Trends in Analytical Chemistry*.

[40] Agrebi S., Larbi A. (2020). Use of Artificial Intelligence in Infectious Diseases. *Artificial Intelligence in Precision Health*.

[41] Parvatikar P., Patil S., Khaparkhuntikar K. (2023). Artificial Intelligence: Machine Learning Approach for Screening Large Database and Drug Discovery. *Antiviral Research*.

[42] Qureshi R., Irfan M., Gondal T. M. (2023). AI in Drug Discovery and its Clinical Relevance. *Heliyon*.

[43] Sharma A., Rani S., Gupta D. (2020). Artificial Intelligence-Based Classification of Chest X-Ray Images Into COVID-19 and Other Infectious Diseases. *International Journal of Biomedical Imaging*.

[44] Kaur S., Singla J., Nkenyereye L. (2020). Medical Diagnostic Systems Using Artificial Intelligence (AI) Algorithms: Principles and Perspectives. *IEEE Access*.

[45] Aiello A., Renson A., Zivich P. (2020). Social Media-and Internet-Based Disease Surveillance for Public Health. *Annual Review of Public Health*.

[46] Lau H. J., Lim C. H., Foo S. C., Tan H. S. (2021). The Role of Artificial Intelligence in the Battle against Antimicrobial-Resistant Bacteria. *Current Genetics*.

[47] Shajari S., Kuruvinashetti K., Komeili A., Sundararaj U. (2023). The Emergence of AI-Based Wearable Sensors for Digital Health Technology: A Review. *Sensors*.

[48] Ueda D., Kakinuma T., Fujita S. (2024). Fairness of Artificial Intelligence in Healthcare: Review and Recommendations. *Japanese Journal of Radiology*.

[49] Khan G. Z., Shah I. A., Hassan M. A., Junaid H., Sardar F. Intelligent Systems for Early Malaria Disease Detection in Patient Cells Using Transfer Learning Approaches.

[50] Equils O., Bakaj A., Wilson-Mifsud B., Chatterjee A. (2023). Restoring Trust: The Need for Precision Medicine in Infectious Diseases, Public Health and Vaccines. *Human Vaccines & Immunotherapeutics*.

[51] Ward R., Aghaeepour N., Bhattacharyya R. (2021). Harnessing the Potential of Multiomics Studies for Precision Medicine in Infectious Disease. *Open Forum Infectious Diseases*.

[52] Marques C., Silva B., Barreto V., Feitoza A., Lira A., Feijão A. (2021). Accuracy of Risk Factors for Nursing Diagnosis Risk of Infection in People With AIDS. *Revista da Escola de Enfermagem da USP*.

[53] Ladner J. T., Grubaugh N. D., Pybus O. G., Andersen K. G. (2019). Precision Epidemiology for Infectious Disease Control. *Nature Medicine*.

[54] Batool M., Galloway-Peña J. (2023). Clinical Metagenomics—Challenges and Future Prospects. *Frontiers in Microbiology*.

[55] Hussein R., Al-Kubaisy S., Al-Ouqaili M. (2024). The Influence of Efflux Pump, Outer Membrane Permeability and β-Lactamase Production on the Resistance Profile of Multi, Extensively and Pandrug Resistant *Klebsiella pneumoniae*. *Journal of Infection and Public Health*.

[56] Conrad S., Kanegusuku A. G., Conklin S. E. (2023). Taking a Step Back From Testing: Preanalytical Considerations in Molecular Infectious Disease Diagnostics. *Clinical Biochemistry*.

[57] Patel R., Tsalik E. L., Evans S., Fowler V. G., Doernberg S. B., Group A. R. L. (2023). Clinically Adjudicated Reference Standards for Evaluation of Infectious Diseases Diagnostics. *Clinical Infectious Diseases*.

[58] Ko E. R., Tsalik E. L. (2022). *A New Era in Host Response Biomarkers to Guide Precision Medicine for Infectious Diseases*.

[59] Roger P., Keïta-Perse O., Mainardi J. (2023). Diagnostic Uncertainty in Infectious Diseases: Advocacy for a Nosological Framework. *Infectious Disease News*.

[60] Saleh R., Al-Ouqaili M., Ali E. (2024). lncRNA-microRNA Axis in Cancer Drug Resistance: Particular Focus on Signaling Pathways. *Medical Oncology*.

[61] Johnson K. B., Wei W. Q., Weeraratne D. (2021). Precision Medicine, AI, and the Future of Personalized Health Care. *Clinical and translational science*.

[62] Ahmed Z., Mohamed K., Zeeshan S., Dong X. (2020). Artificial Intelligence With Multi-Functional Machine Learning Platform Development for Better Healthcare and Precision Medicine. *Database: The Journal of Biological Databases and Curation*.

[63] Qi F., Li J., Qi Z. (2023). Comprehensive Metabolic Profiling and Genome-Wide Analysis Reveal Therapeutic Modalities for Hepatocellular Carcinoma. *Research: Ideas for Today’s Investors*.

[64] Doran J., Thompson R., Yates C., Bowness R. (2023). Mathematical Methods for Scaling From Within-Host to Population-Scale in Infectious Disease Systems. *Epidemics*.

[65] Morrocchi E., van Haren S., Palma P., Levy O. (2023). Modeling Human Immune Responses to Vaccination In Vitro. *Trends in Immunology*.

[66] Kabir R., Syed H. Z., Vinnakota D. (2023). Deep Learning for Clinical Decision-Making and Improved Healthcare Outcome. *Deep Learning in Personalized Healthcare and Decision Support*.

[67] Kaur I., Kumar Y., Sandhu A. K., Ijaz M. F. (2023). Predictive Modeling of Epidemic Diseases Based on Vector-Borne Diseases Using Artificial Intelligence Techniques. *Computational Intelligence in Medical Decision Making and Diagnosis*.

[68] Zheng R., Liu G. (2023). Application of Machine Learning in Clinical Predictive Models for Infectious Diseases: A Review. *Chinese Journal of Schistosomiasis Control*.

[69] Thakur K., Kaur M., Kumar Y. Artificial Intelligence Techniques to Predict the Infectious Diseases: Open Challenges and Research Issues.

[70] Baldominos A., Puello A., Oğul H., Aşuroğlu T., Colomo-Palacios R. (2020). Predicting Infections Using Computational Intelligence–A Systematic Review. *IEEE Access*.

[71] Kurant D. E. (2023). Opportunities and Challenges with Artificial Intelligence in Genomics. *Clinics in Laboratory Medicine*.

[72] Chandrashekar K., Niranjan V., Vishal A., Setlur A. S. (2024). Integration of Artificial Intelligence, Machine Learning and Deep Learning Techniques in Genomics: Review on Computational Perspectives for NGS Analysis of DNA and RNA Seq Data. *Current Bioinformatics*.

[73] Kaushal V., Gupta R. (2022). Role of Artificial Intelligence in Diagnosis of Infectious Diseases. *Biomedical Translational Research: Technologies for Improving Healthcare*.

[74] Alfardan A. A. I., Alharbi R. F. R., Alrammaal W. H. A. (2024). The Role of Artificial Intelligence in Predicting Disease Outbreaks: A Multidisciplinary Approach. *International Journal of Health Sciences*.

[75] Sarantopoulos A., Yokarasa A., Makamanzi C., Antoniou P., Spernovasilis N., Tsioutis C. (2024). Artificial Intelligence in Infectious Disease Clinical Practice: An Overview of Gaps, Opportunities, and Limitations. *Tropical Medicine and Infectious Disease*.

[76] Owaid H. A., Al-Ouqaili M. T. (2025). Molecular Characterization and Genome Sequencing of Selected Highly Resistant Clinical Isolates of *Pseudomonas aeruginosa* and its Association With the Clustered Regularly Interspaced Palindromic Repeat/Cas System. *Heliyon*.

[77] Vladyka R., Skril I. (2023). Using Machine Learning Algorithms to Analyze Genetic Data for Disease Diagnosis. *Collection of Scientific Papers*.

[78] Peiffer-Smadja N., Rawson T. M., Ahmad R. (2020). Machine Learning for Clinical Decision Support in Infectious Diseases: A Narrative Review of Current Applications. *Clinical Microbiology and Infection*.

[79] Egli A., Schrenzel J., Greub G. (2020). Digital Microbiology. *Clinical Microbiology and Infection*.

[80] Kulkarni P. A., Singh H. (2023). Artificial Intelligence in Clinical Diagnosis: Opportunities, Challenges, and Hype. *Journal of the American Medical Association*.

[81] Cheng K., Li Z., He Y. (2023). Potential Use of Artificial Intelligence in Infectious Disease: Take ChatGPT as an Example. *Annals of Biomedical Engineering*.

[82] Tran N. K., Kretsch C., LaValley C., Rashidi H. H. (2023). Machine Learning and Artificial Intelligence for the Diagnosis of Infectious Diseases in Immunocompromised Patients. *Current Opinion in Infectious Diseases*.

[83] Bawa A., Kansal R., Kalra V., Rengan V., Sundaram P., Ramana B. (2023). Oc-004 Can AI Help in Early Prediction of Surgical Site Infections?. *British Journal of Surgery*.

[84] Al Kuwaiti A., Nazer K., Al-Reedy A. (2023). A Review of the Role of Artificial Intelligence in Healthcare. *Journal of Personalized Medicine*.

[85] Huang S., Chaudhari A., Langlotz C., Shah N., Yeung S., Lungren M. (2022). Developing Medical Imaging AI for Emerging Infectious Diseases. *Nature Communications*.

[86] Branda F., Lodi G. (2023). Challenges and Perspectives of Open Data in Modelling Infectious Diseases. *Data*.

[87] Tsueng G., Cano M., Bento J. (2023). Developing a Standardized but Extendable Framework to Increase the Findability of Infectious Disease Datasets. *Scientific Data*.

[88] Kherabi Y., Ming D., Rawson T. M., Peiffer-Smadja N. (2022). Challenges in Implementing Clinical Decision Support Systems for the Management of Infectious Diseases. *Diverse Perspectives and State-of-the-Art Approaches to the Utilization of Data-Driven Clinical Decision Support Systems*.

[89] Chidzwondo F., Mutapi F. (2021). Challenge of Diagnosing Acute Infections in Poor Resource Settings in Africa. *AAS Open Research*.

[90] Sharma A. K., Joshi A. (2022). Impact of AI, IoT and Big Data Analytics in Diseases Diagnosis and Prediction. *Transformation in Healthcare With Emerging Technologies*.

[91] Zhang Q. (2021). Data Science Approaches to Infectious Disease Surveillance. *Philosophical Transactions. Series A, Mathematical, Physical, and Engineering Sciences*.

[92] Obaid A. J. Data Mining Analysis Models Based on Prospective Detection of Infectious Disease.

[93] Becker A. D., Grantz K. H., Hegde S. T., Bérubé S., Cummings D. A., Wesolowski A. (2021). Development and Dissemination of Infectious Disease Dynamic Transmission Models during the COVID-19 Pandemic: What Can We Learn From Other Pathogens and How Can We Move Forward?. *The Lancet Digital Health*.

[94] Shree A. R., Kiran P., Mohith N., Kavya M. (2021). Sensitivity Context Aware Privacy Preserving Disease Prediction. *Expert Clouds and Applications: Proceedings of ICOECA*.

[95] Vuong K., Ivers R., Dykgraaf S. H., Nixon M., Roberts G., Liaw S.-T. (2022). Ethical Considerations Regarding the Use of Pooled Data From Electronic Health Records in General Practice. *Australian Journal of General Practice*.

[96] Chiruvella V., Guddati A. (2021). Ethical Issues in Patient Data Ownership. *Interactive Journal of Medical Research*.

[97] Kitamura K., Irvan M., Shigetomi Yamaguchi R. Disclosure of Multiple Patient Characteristics Format Statistics Leaks Quasi-Identifier Linkage.

[98] Asra’a A., Al-Khafaji Z. M., Al-Ouqaili M. T. (2018). Investigation of Accd3 Gene of Mycobacterium Tuberculosis Iraqi Isolates. *Asian Journal of Pharmaceutical and Clinical Research*.

[99] Zabor E. C., Reddy C. A., Tendulkar R. D., Patil S. (2022). Logistic Regression in Clinical Studies. *International Journal of Radiation Oncology, Biology, Physics*.

[100] Khyathi G., Indumathi K., Hasin J., Siluvai S., Krishnaprakash G. (2025). Support Vector Machines: A Literature Review on Their Application in Analyzing Mass Data for Public Health. *Cureus*.

[101] Siddiqui E. F., Ahmed T., Nayak S. K. (2024). A Decision Tree Approach for Enhancing Real-Time Response in Exigent Healthcare Unit Using Edge Computing. *Measurement: Sensors*.

[102] Chaw J. K., Chaw S. H., Quah C. H. (2024). A Predictive Analytics Model Using Machine Learning Algorithms to Estimate the Risk of Shock Development Among Dengue Patients. *Healthcare Analytics*.

[103] Mishra S., Kumar R., Tiwari S. K., Ranjan P. (2022). Machine Learning Approaches in the Diagnosis of Infectious Diseases: a Review. *Bulletin of Electrical Engineering and Informatics*.

[104] Zaeri N. (2023). Artificial Intelligence and Machine Learning Responses to COVID-19 Related Inquiries. *Journal of Medical Engineering & Technology*.

[105] Yalew G., Muthupandian S., Hagos K. (2022). Prevalence of Bacterial Vaginosis and Aerobic Vaginitis and Their Associated Risk Factors Among Pregnant Women From Northern Ethiopia: A Cross-Sectional Study. *PLoS One*.

[106] Ejikeugwu C., Nworie O., Saki M. (2021). Metallo-β-Lactamase and AmpC Genes in *Escherichia coli*, *Klebsiella pneumoniae*, and *Pseudomonas aeruginosa* Isolates From Abattoir and Poultry Origin in Nigeria. *BMC Microbiology*.

[107] Geric C., Qin Z., Denkinger C. (2023). The Rise of Artificial Intelligence Reading of Chest X-Rays for Enhanced TB Diagnosis and Elimination. *International Journal of Tuberculosis & Lung Disease*.

[108] Rudnicka Z., Szczepanski J., Pregowska A. (2024). Artificial Intelligence-Based Algorithms in Medical Image Scan Segmentation and Intelligent Visual Content Generation—A Concise Overview. *Electronics*.

[109] Chen Q. (2024). Computational Linguistics and Biological Sequences in Artificial Intelligence. *Association Analysis Techniques and Applications in Bioinformatics*.

[110] Ibrahim A. U., Ozsoz M., Serte S., Al-Turjman F., Yakoi P. S. (2024). Pneumonia Classification Using Deep Learning From Chest X-Ray Images During COVID-19. *Cognitive Computation*.

[111] Al-qaness M. A., Zhu J., Al-Alimi D. (2024). Chest X-Ray Images for Lung Disease Detection Using Deep Learning Techniques: A Comprehensive Survey. *Archives of Computational Methods in Engineering*.

[112] Ijeh S., Okolo C. A., Arowoogun J. O., Adeniyi A. O., Omotayo O. (2024). Predictive Modeling for Disease Outbreaks: A Review of Data Sources and Accuracy. *International Medical Science Research Journal*.

[113] Rafique Q., Rehman A., Afghan M. S. (2023). Reviewing Methods of Deep Learning for Diagnosing COVID-19, Its Variants and Synergistic Medicine Combinations. *Computers in Biology and Medicine*.

[114] Gao Y., Liu M. (2024). Application of Machine Learning Based Genome Sequence Analysis in Pathogen Identification. *Frontiers in Microbiology*.

[115] Struelens M. J., Ludden C., Werner G., Sintchenko V., Jokelainen P., Ip M. (2024). Real-Time Genomic Surveillance for Enhanced Control of Infectious Diseases and Antimicrobial Resistance. *Frontiers in Science*.

[116] Harris M. (2023). Machine Learning and Artificial Intelligence for Pathogen Identification and Antibiotic Resistance Detection: Advancing Diagnostics for Urinary Tract Infections. *Biology*.

[117] Tripathi A., Rathore R. (2025). AI in Disease Surveillance—An Overview of How AI Can Be Used in Disease Surveillance and Outbreak Detection in Real‐World Scenarios. *Disease Detection: Advancements and Applications*.

[118] Zahra M. A., Al-Taher A., Alquhaidan M. (2024). The Synergy of Artificial Intelligence and Personalized Medicine for the Enhanced Diagnosis, Treatment, and Prevention of Disease. *Drug Metabolism and Personalized Therapy*.

[119] Almotairi K. H., Hussein A. M., Abualigah L. (2023). Impact of Artificial Intelligence on COVID-19 Pandemic: A Survey of Image Processing, Tracking of Disease, Prediction of Outcomes, and Computational Medicine. *Big Data and Cognitive Computing*.

[120] Dhade P., Shirke P. (2024). Federated Learning for Healthcare: A Comprehensive Review. *Engineering Proceedings*.

[121] Khezr S., Moniruzzaman M., Yassine A., Benlamri R. (2019). Blockchain Technology in Healthcare: A Comprehensive Review and Directions for Future Research. *Applied Sciences*.

[122] Moon G., Yang Jh, Son Y., Choi Ek, Lee I. (2023). Ethical Principles and Considerations Concerning the Use of Artificial Intelligence in Healthcare. *Korean Journal of Medical Ethics*.

[123] Kour H., Kulkarni S. L. (2024). Ethical Considerations in the Use of Artificial Intelligence in Health Care With Insights From the Indian Context. *Journal of the Scientific Society*.

[124] Tamò‐Larrieux A., Guitton C., Mayer S., Lutz C. (2024). Regulating for Trust: Can Law Establish Trust in Artificial Intelligence?. *Regulation & Governance*.

[125] Al-Hwsali A., Alsaadi B., Abdi N. (2023). Scoping Review: Legal and Ethical Principles of Artificial Intelligence in Public Health. *Studies in Health Technology and Informatics*.

[126] Bolton W. J., Badea C., Georgiou P., Holmes A., Rawson T. M. Developing Moral AI to Support Antimicrobial Decision Making. https://arxiv.org/abs/2208.06327.

[127] Zhu H., Li Y., Zhu D. (2024). Establishment and Application of an Artificial Intelligence-Assisted Platform for Detection of Parasite Eggs. *Chinese Journal of Schistosomiasis Control*.

[128] Herath H., Herath H., Madhusanka B., Guruge L. (2024). Data Protection Challenges in the Processing of Sensitive Data. *Data Protection: The Wake of AI and Machine Learning*.

[129] Khan M. S., Umer H., Faruqe F. (2024). Artificial Intelligence for Low Income Countries. *Humanities and Social Sciences Communications*.

[130] Amin S., El-Gazar H., Zoromba M., El-Sayed M., Atta M. (2024). Sentiment of Nurses Towards Artificial Intelligence and Resistance to Change in Healthcare Organisations: A Mixed-Method Study. *Journal of Advanced Nursing*.

[131] Zaidan E., Ibrahim I. A. (2024). AI Governance in a Complex and Rapidly Changing Regulatory Landscape: A Global Perspective. *Humanities and Social Sciences Communications*.

[132] He J., Baxter S. L., Xu J., Xu J., Zhou X., Zhang K. (2019). The Practical Implementation of Artificial Intelligence Technologies in Medicine. *Nature Medicine*.

[133] Zeng D., Cao Z., Neill D. (2020). Artificial Intelligence–Enabled Public Health Surveillance—From Local Detection to Global Epidemic Monitoring and Control. *Artificial Intelligence in Medicine*.

[134] Abdulkareem M., Petersen S. E. (2021). The Promise of AI in Detection, Diagnosis, and Epidemiology for Combating COVID-19: Beyond the Hype. *Frontiers in Artificial Intelligence*.

[135] Pun F., Ozerov I., Zhavoronkov A. (2023). AI-Powered Therapeutic Target Discovery. *Trends in Pharmacological Sciences*.

[136] Gulfidan G., Beklen H., Arga K. (2021). Artificial Intelligence as Accelerator for Genomic Medicine and Planetary Health. *OMICS: A Journal of Integrative Biology*.

[137] Jones-Jang S. M., Park Y. J. (2023). How Do People React to AI Failure? Automation Bias, Algorithmic Aversion, and Perceived Controllability. *Journal of Computer-Mediated Communication*.

[138] Hassija V., Chamola V., Mahapatra A. (2023). Interpreting Black-Box Models: A Review on Explainable Artificial Intelligence. *Cognitive Computation*.

[139] Albahri A., Duhaim A. M., Fadhel M. A. (2023). A Systematic Review of Trustworthy and Explainable Artificial Intelligence in Healthcare: Assessment of Quality, Bias Risk, and Data Fusion. *Information Fusion*.

